# 

**Published:** 1906-06

**Authors:** 


**SUBSCRIPTION $1.00.**

**PUBLISHED MONTHLY.**

### ATLANTA JOURNAL-RECORD OF MEDICINE.

JUN. 15 1906

**Successor to Atlanta Medical and Surgical Journal, Established 1855, and Southern Medical Record, Established 1870.**

VOL. VIII. ATLANTA, GA., JUNE, 1906. NO. 3.

